# Oguchi’s disease with Mizuo-Nakamura phenomenon in a seven-year-old boy

**DOI:** 10.3205/oc000089

**Published:** 2018-12-13

**Authors:** Pukhraj Rishi, Ekta Rishi, Sharanya Abraham

**Affiliations:** 1 Shri Bhagwan Mahavir Vitreoretinal Services, Sankara Nethralaya, Chennai, India

## Abstract

A seven-year-old boy presented with difficulty in night vision of five years duration. Vision was 20/20 OU. Fundus examination revealed a golden sheen over the posterior pole in both eyes which disappeared after 45 minutes of dark adaptation, suggestive of Mizuo-Nakamura phenomenon. Clinical findings were suggestive of Oguchi’s disease. ERG was confirmatory.

## Case description

A seven-year-old boy presented with a history of difficulty in night vision for the past five years. He was the older twin, born at term with normal birth history and developmental milestones. Family history was unremarkable and physical examination was normal. His best corrected visual acuity was 20/20 in both eyes. The anterior segment examination was normal in both eyes. The other twin had no ocular complaints and his fundus examination was unremarkable.

**Figure 1:**
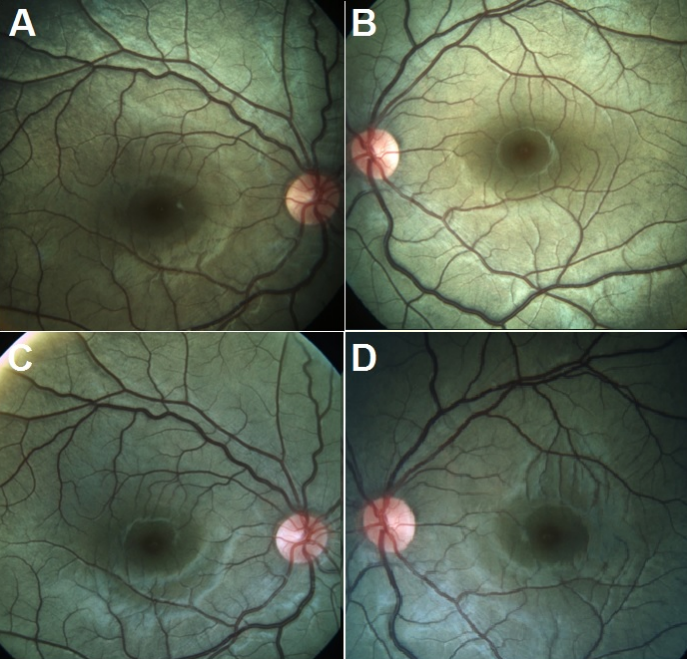
Fundus photos of the right (A) and left (B) eye reveal a golden sheen caused by the Mizuo-Nakamura phenomenon. The golden sheen is extinguished after 45 minutes of dark adaptation as seen in the fundus photos of the right (C) and left (D) eye.

**Figure 2:**
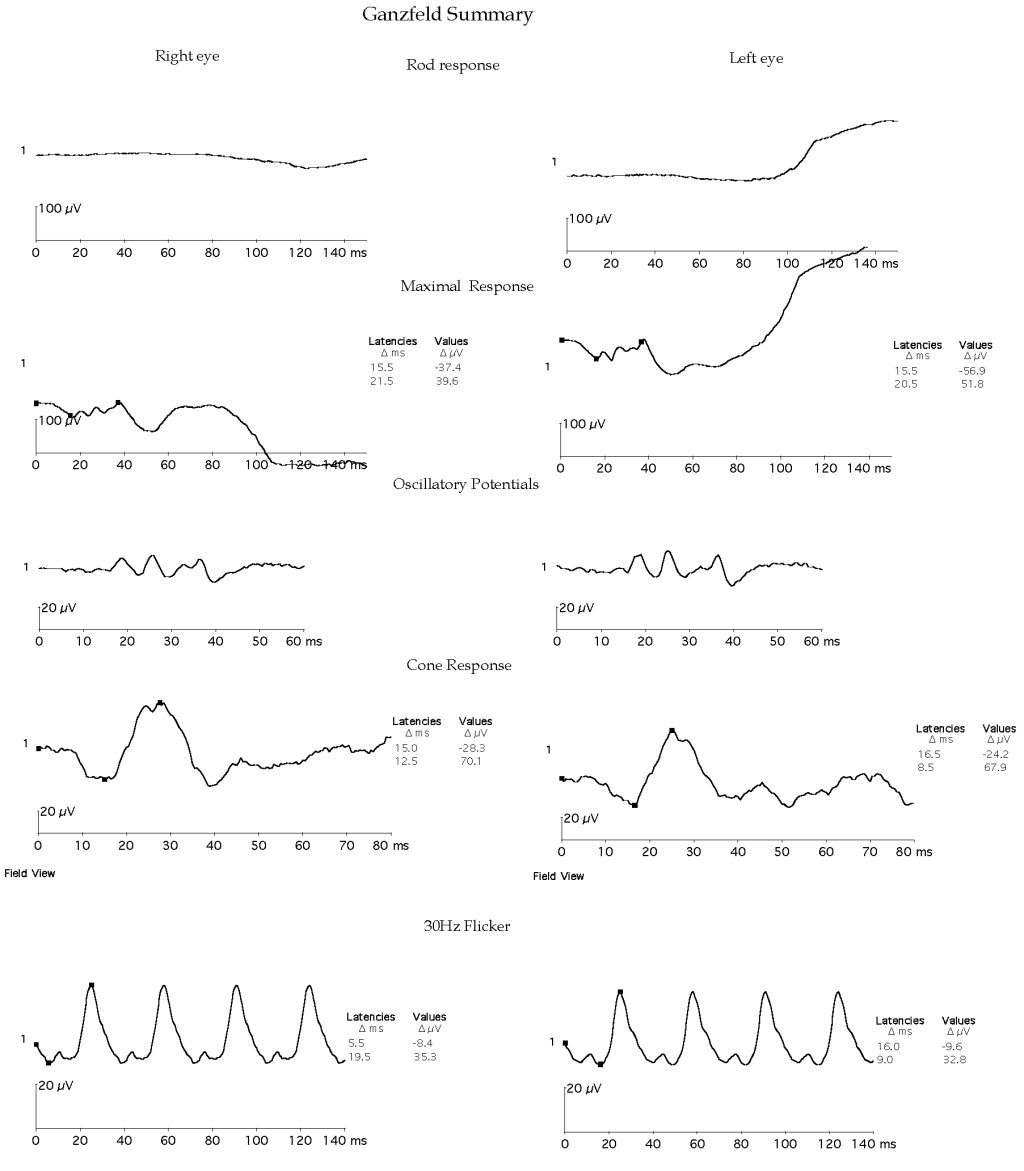
ERG showing the negative waveform morphology with non-recordable single flash rod response, negative-positive combined response, and normal photopic response

Dilated fundus examination of both eyes revealed a golden sheen over the posterior pole, suggestive of the Mizuo-Nakamura phenomenon (Figure 1A,B (Fig. 1)). This sheen disappeared after 45 minutes of dark adaptation (Figure 1C,D (Fig. 1)). There was no evidence of bone-spicules pigmentation and the optic disc and arterioles were normal. A clinical diagnosis of Oguchi’s disease was arrived at. The electroretinogram (ERG) of both eyes showed the characteristic negative waveform morphology with non-recordable single flash rod response, negative-positive combined response and normal photopic response (Figure 2 (Fig. 2)), thereby confirming the clinical diagnosis. The nature of the disease and need for family screening were explained. Genetic testing was offered but declined by the family. At ten-month follow-up, his visual acuity, ocular and ERG findings were stable compared to the first visit.

## Discussion

Oguchi’s disease is an autosomal recessive form of congenital stationary night blindness and is caused by a disruption in the steps of inactivation of rod phototransduction. Patients with this disease often complain of static nyctalopia since childhood and a preservation of vision in bright light. Rhodopsin kinase (GRK1) and Arrestin (SAG) are two genes which act in sequence to deactivate rhodopsin to stop the phototransduction cascade [1]. Photoactivated rhodopsin is recognized by rhodopsin kinase, which phosphorylates serine and threonine residues near rhodopsin’s carboxy terminus. Arrestin then forms a complex with phosphorylated rhodopsin, and this complex prevents further interaction of the activated rhodopsin with transducin. Mutations in either the rhodopsin kinase gene or the arrestin gene cause a recessive form of stationary night blindness called Oguchi’s disease. As the mutation is recessive, either there are no gene products or the resultant mutant proteins are inactive. 

The dark adaptation curve in patients with Oguchi’s disease is distinctly prolonged, while the early cone branch of the curve is normal. Sensitivity plateaus at the cone level for 1 to 2 hours. At this time a rod-cone break occurs and there is full recovery of rod sensitivity over the next 1 to 2 hours. This causes the characteristic ERG findings, which after another prolonged period of dark adaptation may revert to normal amplitude and timing [1], [2]. The recovery of light sensitivity in Oguchi’s disease is related to the time taken for rhodopsin regeneration. Light adaptation causes millions of rhodopsin molecules within a rod to be bleached. In a normal retina, these bleached molecules are quickly inactivated by rhodopsin kinase and arrestin. The abnormal genetic function in Oguchi’s disease results in the continuous activation of transducin by photoactivated rhodopsin, thereby desensitizing the rods. Patients with Oguchi’s disease must wait for complete regeneration of almost all of their rhodopsin molecules before the rods regain full sensitivity [1]. This process may take more than two hours.

The other distinctive feature of Oguchi’s disease is that while the colour of the fundus is normal after prolonged dark adaptation, it changes to a dark or golden hue after a few minutes in the light. The colour change is called the Mizuo phenomenon or the Mizuo-Nakamura phenomenon and its biochemical basis is not known. It is speculated to be the result of elevated extracellular potassium levels generated in the retina in response to an excessive stimulation of rod photoreceptors [3]. 

Reports in literature have suggested that patients with mutations in the rhodopsin kinase gene have Oguchi’s disease with no signs of photoreceptor degeneration, while those with arrestin mutations have some features of Oguchi’s disease, such as the Mizuo-Nakamura phenomenon [4].

In at least one family, two affected siblings homozygous for the same arrestin gene defect differed in their phenotypes: one had classic Oguchi’s disease, the other retinitis pigmentosa [5]. However, the cause as to why some patients with Oguchi’s disease develop retinitis pigmentosa remains unknown. In addition to Oguchi’s disease, the Mizuo-Nakamura phenomenon and similar tapetal-fundus reflexes have also been reported in retinitis pigmentosa [6], X-linked retinoschisis [3], and cone-rod dystrophy [7]. Of late, there have been reports in the lay press on the possible role of Zinc in the treatment of Oguchi’s disease. However, these appear to be speculative – Pubmed search with the keywords (Oguchi’s disease) and (Zinc) did not reveal any published reports. It’s noteworthy that Zinc deficiency may cause abnormal dark adaptation as it is required for vitamin A metabolism. 

In conclusion, Oguchi’s disease presents with clinical features which are characteristic but likely to be missed if not correlated with a relevant history and electrophysiological testing. The combination of normal cone function, delayed rod ERG dark adaptation, and marked rod desensitization to a bright flash is distinctive for Oguchi’s disease. Where necessary, the disease may be documented based on genetic testing. 

## Notes

### Competing interests

The authors declare that they have no competing interests.
